# Data from quality life questionnaries: QLQ-C30 and QLQ-BR23 in a cohort of Women with breast cancer in Cali, Colombia - 2020

**DOI:** 10.1016/j.dib.2021.106878

**Published:** 2021-02-24

**Authors:** Mauricio Hernández-Carrillo, María Elena Mejía Rojas, Adolfo Contreras Rengifo, Edgar Muñoz

**Affiliations:** a Doctorate in Health, Universidad del Valle, Cali, Colombia; b School of Nursing, Nursing Care Research Group, Universidad del Valle, Cali, Colombia; c School of Dentistry, Periodontal Medicine Research Group, Universidad del Valle, Cali, Colombia; d University of Texas Health Science Center at San Antonio, San Antonio, TX, USA

**Keywords:** Breast cancer, Health-related quality of life, Chemotherapy, Cancer pain, Women's health, Colombia (MeSH)

## Abstract

In an attempt to evaluate the contribution of symptoms, functionality and health perception on health-related quality of life of 80 women with breast cancer that underwent chemotherapy for disease treatment was studied. In this study cohort, retrospective data was extracted after the application of the questionnaires QLQ-C30 and QLQ-BR23 for quality of life. These patients belong 3 oncology centers in Cali – Colombia 2020. The quality of life was determined by measuring the interaction of 3 components represented by the global health status, the functional status and the symptoms.

## Specifications Table

**Table:** 

Subject	Health and medical sciences
Specific subject area	Oncology
Type of data	Table
How data were acquired	The data were collected with the application of two questionnaires QLQ-C30 (European organization for research and treatment of cancer quality life questionnaire core 30) and QLQ-BR23 (quality life questionnaire for breast cancer specific-questionnaire) to determine the quality of life in women with breast cancer in the city of Cali - Colombia.
Data format	Raw data
Parameters for data collection	385 women with breast cancer was reported in Cali in 2016. Based on information from individual service delivery records, 275 patients were receiving chemotherapy as part of its disease treatment. The sample frame was 187 patients, however only 80 patients accepted to participate in this study. The distribution of women by institution was as follows: 53 subjects from Hospital Universitario del Valle; 18 from Centro Médico Imbanaco; and finally 9 patients from Funcancer. One of the center was public and the other 2, private.
Description of data collection	Data collection was conducted between July 2017 and June 2018. Three questionnaires were applied to the patients: the first one to recall sociodemographic data, the second was the EORTC (European Organization for Research and Treatment of Cancer) quality of life scales QLQ-C30 and the last one was specific for breast cancer EORTC QLQ-BR23 and including psychometric tests validated for Colombia.
Data source location	Institution: Universidad del Valle City/Town/Region: Cali/Valle del Cauca Country: Colombia
Data accessibility	Repository name: Mendeley Data Data identification number: https://doi.org/10.17632/p4cnd87hxh.3 Direct URL to data: https://data.mendeley.com/datasets/p4cnd87hxh/3
Related research article	Mejía-Rojas ME, Contreras-Rengifo A, HernándezCarrillo M. Calidad de vida en mujeres con cáncer de mama sometidas a quimioterapia en Cali, Colombia. Biomédica. 2020;40:349-61. https://doi.org/10.7705/biomedica.4971

## Value of the Data

• The data is aimed to design interventions for improving the living conditions of women with breast cancer that underwent chemotherapy. Therefore, the findings could be extrapolated to other groups having Breast Cancer because, QLQ-C30 (European organization for research and treatment of cancer quality life questionnaire core 30) and the QLQ-BR23 (quality life questionnaire Breast cancer specific-questionnaire), are commonly used instruments for measuring quality of life in oncology.
• Breast cancer treatment comply is associated to social, cultural, chemotheraphy side effects and other familiar and medical factors and could vary between geographic regions or countries. Then, public health policies and treatment guidelines must take in consideration those complex aspects associated to life quality.
• Researchers in the field of oncology could integrate this specific Colombian breast cancer data into more huge data bases for further analysis including another Latin American information that is always difficult to recall to expand the knowledge.
• As these data are raw, they can be used to understand the phenomenon of quality of life in breast cancer, and can also be used to perform univariate, bivariate and multivariate analyses and reveal biological and society and cultural differences among diverse populations.
• This data sharing might impact society for the improvement of the quality of life of women with breast cancer at health care services.
• Finally, this data can help to develop strategic decision-making regarding the implementation and adjustment of public health policies on breast cancer and cancer-related quality of life.

## 1 Data Description

Raw data: describes the assessment of general quality of life and health-related quality of life in women with breast cancer in the QLQ-C30 and QLQ-BR23 questionnaires, in the observable variables: symptoms, functionality and health perception, according to the method described in the EORTC (European Organisation for Research and Treatment of Cancer) manual for QLQ [1].

The cancer questionnaire in general (QLQ-C30) evaluated 30 items, while the QLQ-BR23 included 23 items. The items are graded by using a Likert scale per domain ranging from 0 to 100, where a high value indicates greater perception of the factor.

### 1.1 Socio-demographic variables

Age in completed years, number of children, marital status of the patient, socioeconomic status, educational level, employment and occupation, and health affiliation were recalled.

### 1.2 Clinical and health service related variables

Institution type (public or private), diagnosis time in months, lawsuit for receiving proper treatment, family history records for breast cancer, breast cancer progression, radiotherapy performed, surgery performed to the patient, tumor stage, chemotherapy regime including tamoxifen and its dosis, disease cycle, original histopathological classification, estrogen receptors detection like having HER2, BRCA and Fish presence, tumor clinical classification at diagnosis time, Karnofsky index and other comorbidities presence.

## 2 Experimental Design, Materials and Methods

Cross-sectional observational research with analytical focus. The target population was women with breast cancer who received chemotherapy in Cali. This study was conducted from secondary information of the health-related quality of life (HRQL) collected from a sample of female patients with breast cancer undergoing chemotherapy treated in three health institutions in Cali - Colombia, from secondary source of data from a baseline study conducted by the Nursing Care group of the University of Valle and funded by the same institution.

The study universe was constituted by the women survivors of breast cancer in Cali (southwestern Colombia), who underwent chemotherapy treatment in a public hospital and two private health institutions in the city of Cali. According to the municipal health secretariat of Cali, 385 cases of breast cancer were reported during 2016, cases identified from the card with code 155 of the National Institute of Health. According to information from the RIPS (individual service delivery records) from the procedure file 275 patients were identified with chemotherapy treatment.

At the participating institutions, 68% of the patients underwent chemotherapy in Cali was identified and a sample frame of 187 patients was calculated. Based on the information obtained from the base study just, 80 patients included. The QLQ-C30 and QLQ-BR23 questionnaires was applied to those patients by experienced and professional nurses one week after chemotherapy treatment. The distribution of participants by institution was as follows: 53 from Hospital Universitario del Valle, 18 from Centro Médico Imbanaco and 9 from Funcancer.

## Ethics Statement

The personal information contained in the database is confidential by using the program AxCrypt - File Security Made Easy version 2.1.1532, which allows for the encryption of patient data. Additionally, the database is be stored in the personal computer of the principal investigator and by the director of the doctoral team work. Both files will be password-protected, ensuring their security.

The present research are be carried out using the data obtained from a previous study called: “quality of life in women with breast cancer under chemotherapy” with internal code number 138-014, which was funded by the Universidad del Valle to the Nursing Care research group, assigned to Center for the Development and Evaluation of Public Health Policy and Technology (CEDETES by its Spanish acronym); therefore the letter of endorsement from the Institutional Human Ethics Review Committee (CIREH by its Spanish acronym), with act number 012-014 for this reference study. A voluntary informed consent was sign by all participants.

## Declaration of Competing Interest

The authors declare that they have no known competing financial interests or personal relationships which have or could be perceived to have influenced the work reported in this article.

## Appendix Supplementary materials

Image, application 1

Image, application 2

Image, application 3

Image, application 4
