# Supplementary figures and images for: Data from quality life questionnaries: QLQ-C30 and QLQ-BR23 in a cohort of Women with breast cancer in Cali, Colombia - 2020
Source: Data Brief. 2021 Feb 24;35:106878. doi: 10.1016/j.dib.2021.106878 (PMC7941080; doi:10.1016/j.dib.2021.106878)

**Ítems QLQ-C30**


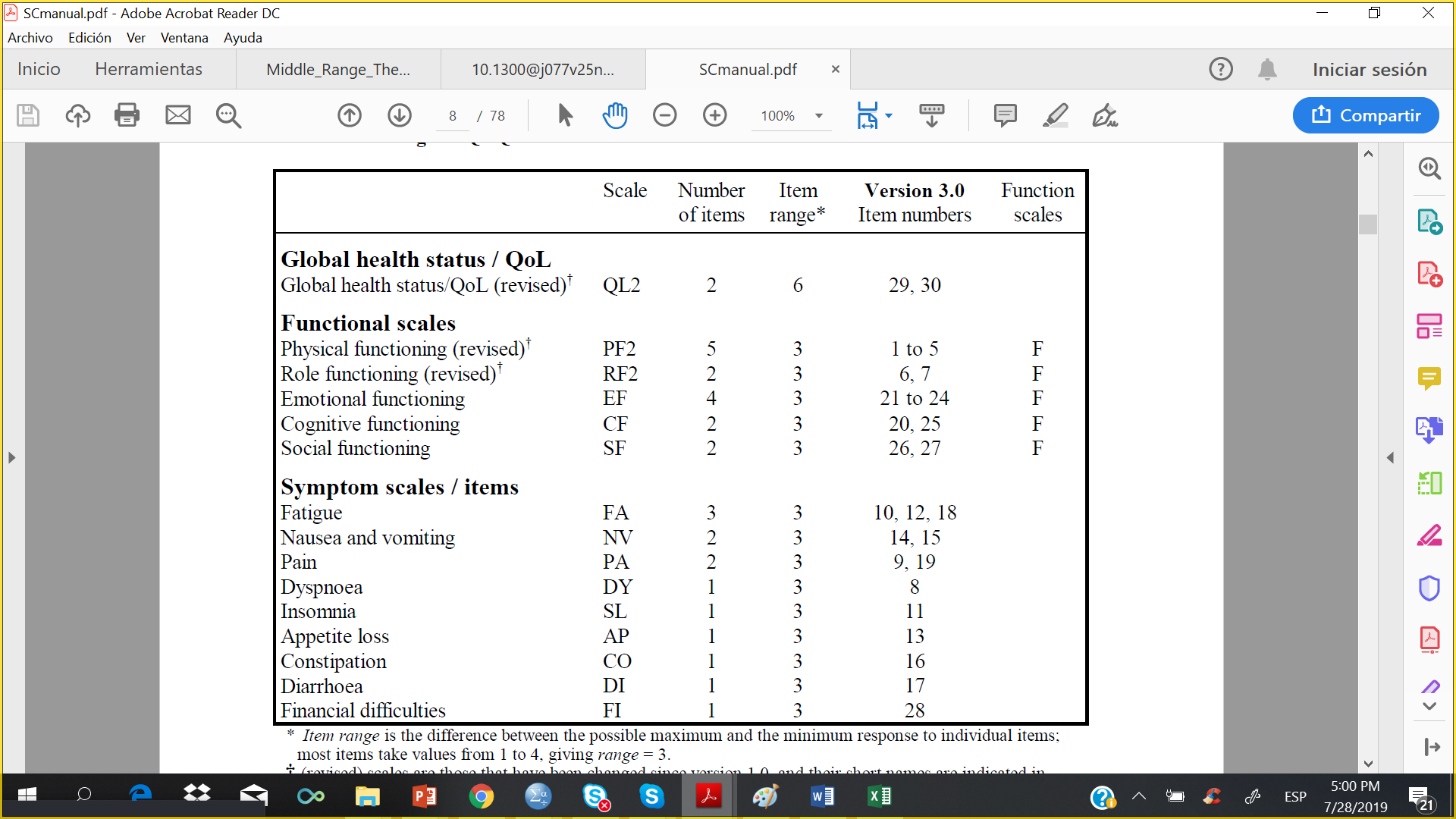


**Ítems QLQ-BR23**


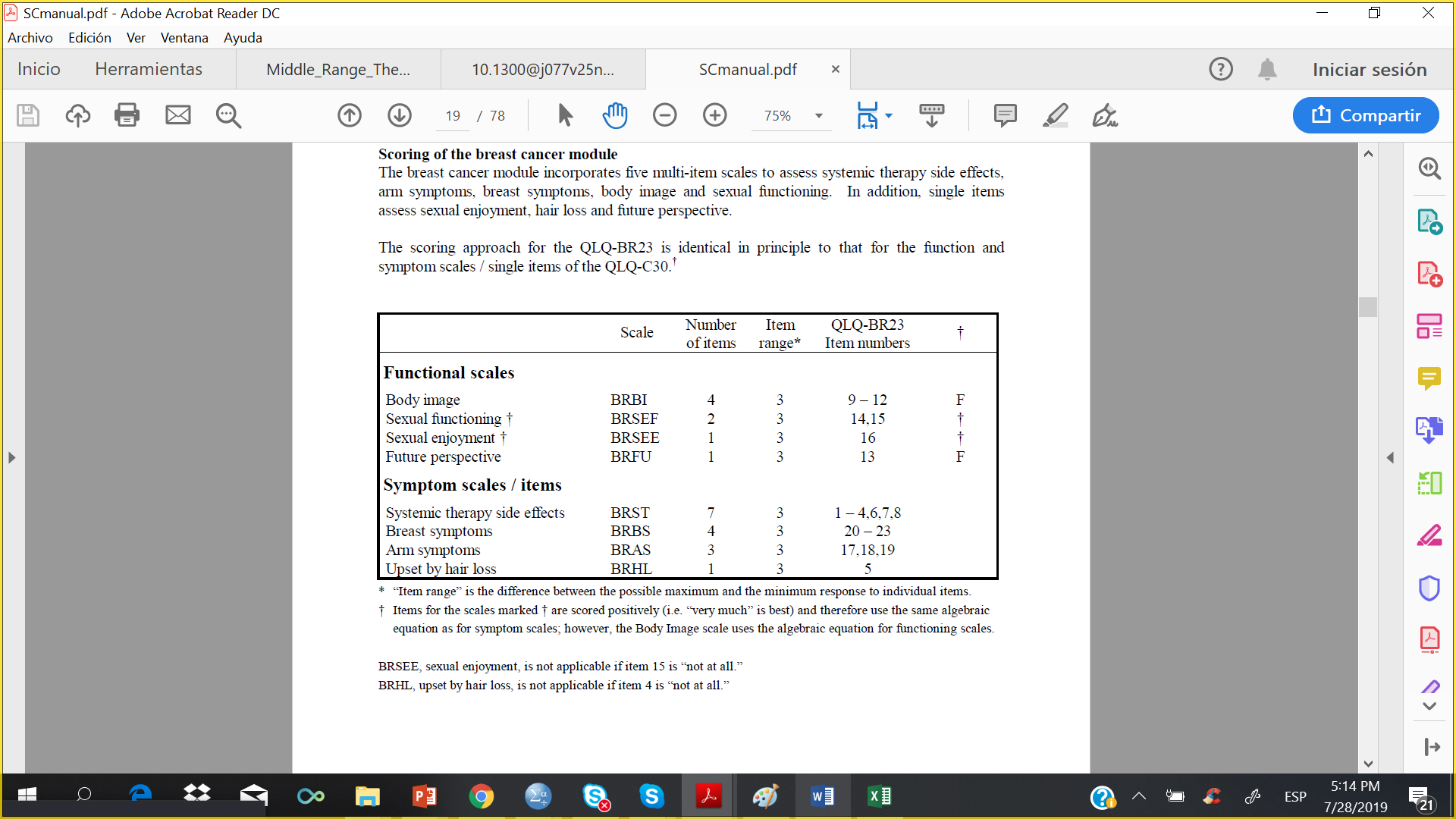

Supplement: Supplementary file 3 [file mmc3.docx]
